# Data on the uptake of Integrated Pest Management (IPM) practices in European agriculture

**DOI:** 10.1016/j.dib.2026.113075

**Published:** 2026-07-13

**Authors:** Sharmin Akter, Cordelia Kreft, Chloe McCallum, Niklas Möhring, Andri Spirig, Erwin Wauters, Clara Sciffer, Vasiliki Inglezou, Georgios Dimitroulas, Silke Dachbrodt-Saaydeh, Lars Ole Hingst, Maria Toader, Cristina Radu, Juan Sagarna, Patricia de Almandoz, Joanna Golian, Joanna Pulawska, Dominika Boguszewska-Mańkowska, Milena Pietraszko, Dorota Labanowska-Bury, Hanka Sobczyńska, Alberto Sturla, Laura Viganò, Maria Grazia Tommasini, Mattia Dall’Ara, Carolina Gattorno, Frans Geijs, Willem Hendriks, Yaite Cuesta Arenas, Harrie Pijnenburgh, Sophie Louise Appel, Jens Erik Jensen, Erato Lazarou, Stella Bitsika, Robert Finger

**Affiliations:** a Agricultural Economics and Policy Group, ETH Zurich, Sonneggstrasse 33, 8092 Zurich, Switzerland; b Queen’s Business School, Queen’s University Belfast, 185 Stranmills Road, Belfast, Northern Ireland, United Kingdom; c Production Economics Group, University of Bonn, Meckenheimer Allee 174, 53115 Bonn, Germany; d Flanders Research Institute for Agriculture, Fisheries and Food (ILVO), Social Sciences Unit, Merelbeke-Melle, Belgium; e NILEAS-Agricultural Olive Oil Cooperative, AgiouDimitriou 36, Chora Messinia, 24600, Greece; f Julius Kühn Institute, Institute for Strategies and Impact Assessment, Stahnsdorfer Damm 81, 14532 Kleinmachnow, Germany; g Crops Sciences Department Faculty of Agriculture, University of Agronomic Sciences and Veterinary Medicine of Bucharest, The Forum of Professional Farmers and Processors from Romania, Romania; h Cooperativas Agro-alimentarias de España, C/Agustín de Betancourt, 17, 4ª Planta 28003, Madrid ES, Spain; i The National Institute of Horticultural Research, Konstytucji 3 Maja 1/3, 96-100 Skierniewice, Poland; j Potato Agronomy Department, Plant Breeding and Acclimatization Institute (IHAR) - National Research Institute, Radzikow, 05-870 Blonie, Poland; k Horticultural Cooperative in Grójec, Mogielnicka 28, 05-600 Grójec, Poland; l Council for Agricultural Research and Economics (CREA), Via Barberini 36 - 00187 Rome, Italy; m Ri.Nova Cooperative Society, Via dell'Arrigoni, 120, 47522 Cesena (FC), Italy; n Southern Agricultural and Horticultural Organization (ZLTO), Onderwijsboulevard 225, 5223 DE 's‑Hertogenbosch, Postbus 100, 5201 AC 's‑Hertogenbosch, the Netherlands; o SEGES Innovation, Agro Food Park 15 DK-8200 Aarhus, Denmark; p Agricultural University of Athens (AUA), Greece; q Department of Agricultural Statistics, Sylhet Agricultural Uinversity, Sylhet-3100, Bangladesh

**Keywords:** Sustainable plant protection, Integrated Pest Management (IPM), Pesticide reduction, Behavioural factors, European agriculture

## Abstract

We here present data collected in a large-scale survey from 25 National Crop Clusters (NCCs) across 10 European countries (Denmark, Germany, Italy, the Netherlands, Belgium, Poland, Romania, Spain, Switzerland, and Greece), comprising 8 different crops (grapes, olives, apples, strawberries, wheat, maize, potatoes, and onions). Each National Crop Cluster represents a crop-specific network in one country, which includes farmers growing a specific crop and other relevant actors in its production environment and supply chain. The National Crop Clusters have been selected to represent a diversity of regions, crops, farm types, sectors and system representatives of European agriculture. They cover a broad geographical spread in different climatic zones, making them suitable for studying Integrated Pest Management (IPM) techniques, barriers, and opportunities in various food sectors. In total, 4,684 complete responses were obtained from the farmers, using an online survey in country-specific languages in 2024. The survey focuses on decisions regarding the adoption of IPM practices and low-pesticide use. The survey data contains information on farmers current and future use of IPM measures, as well as their perceived effectiveness. The dataset further provides comprehensive information on farm and farmer characteristics, farmers’ preferences and attitudes toward IPM, their sources of information, and perceived barriers to adoption. Additionally, data were collected regarding behavioural factors such as non-cognitive skills and risk preferences. This harmonized and broad coverage survey data are particularly useful to identify factors facilitating or hindering the uptake of IPM measures, considering the heterogeneity of farmer’s behaviour, socio-economic and environmental factors in European agriculture. Nevertheless, sample sizes differ across the national crop clusters, and the dataset is not fully representative for some national crop clusters. In addition, for several national crop clusters the share of organic farmers in the datasets is higher than the national average, which shall be considered when interpreting responses related to crop protection practices, IPM uptake, and information sources.

Specifications Table

**Table:** 

Subject	Social Sciences
Specific subject area	Farmers’ uptake of Integrated Pest Management (IPM) practices and their drivers*.*
Type of data	Tables (.csv format), supporting material (codebook and text version of survey questionnaires in English) Data format: Raw and partly filtered for reasons of confidentiality
Data collection	Data were collected using an online questionnaire distributed to over 40000 grain, fruit, and vegetable producers in 10 countries (Denmark, Germany, Italy, Netherlands, Belgium, Poland, Romania, Spain, Switzerland, Greece) producing 8 crops (in different production systems: grapes, olives, apples, strawberries, wheat, maize, potatoes, onions). Data were collected in 25 crop-country combinations, called National Crop Clusters (NCCs), see table 1 below. The questionnaires were identical across National Crop Clusters except for some crop specific IPM measures and country specific labels as well as agri-environmental schemes^1^. The links to the survey were sent to farmers via email directly for Switzerland and Belgium (in Flanders, the Northern part of Belgium), and other National Crop Clusters were approaching the farmers through different channels e.g., farmer associations, cooperative websites, newsletters, magazines, social media campaigns, and specialized events. In seven countries, data were additionally collected by a market research agency. The survey was open for Switzerland from March 12 to June 5, 2024, for National Crop Cluster olive Greece was open from March 22 to July 12, 2024, and for the rest of the National Crop Clusters between March and December 2024. In total, 4,684 farmers fully answered the questionnaire (total N: 4,684). Participation was incentivised^2^. The data were anonymized.
Data source location	Denmark, Germany, Italy, Netherlands, Belgium, Poland, Romania, Spain, Switzerland, and Greece.
Data accessibility	Repository name: ETH research collection Data identification number: https://doi.org/10.3929/ethz-c-000796959
Related research article	None

1 The questionnaire for Swiss farmers did not contain questions on farm structural variables (e.g. farm size) since the survey data could be matched with official farm census data.

2 Across all National Crop Clusters, participants could additionally opt to receive a short feedback report summarizing the survey findings. In addition, in Switzerland, survey participation was incentivized by a lottery of 25 vouchers (50 CHF each). In Belgium, respondents could win 20 vouchers (€25 each) and 5 vouchers (€100 each).

## 1 Value of the Data

• This dataset provides harmonized survey data on the current uptake of IPM practices across European agriculture, covering 8 crops and 10 countries, and both annual and perennial systems
• With an extensive sample size (N = 4,684), the dataset offers a broad empirical basis for comparative analyses across crops, countries, and production systems
• The data can be used to investigate explanatory factors of the implementation of IPM measures and enable to better understand the dynamics of pesticide reduction in European agriculture.
• The dataset includes a broad range of variables on farmers’ attitudes, perceptions, individual preferences, and other behavioural factors. These data can be used by researchers and decision makers to study drivers and barriers of farmers’ IPM adoption to ultimately pave the way for an effective reduction of pesticide use and risk in agriculture.
• The data also can be used to compare farmer characteristics such as behavioural factors, for example in individual participant meta-analysis. The data allow the analysis of farmers’ information sources and their trust in different advisory channels for crop protection practices to find effective ways to communicate and share knowledge in agriculture. In addition, the dataset supports comparisons of the current uptake of IPM measures, as well as their drivers and barriers, across European agriculture.

## 2 Background

**Fig. 1:**
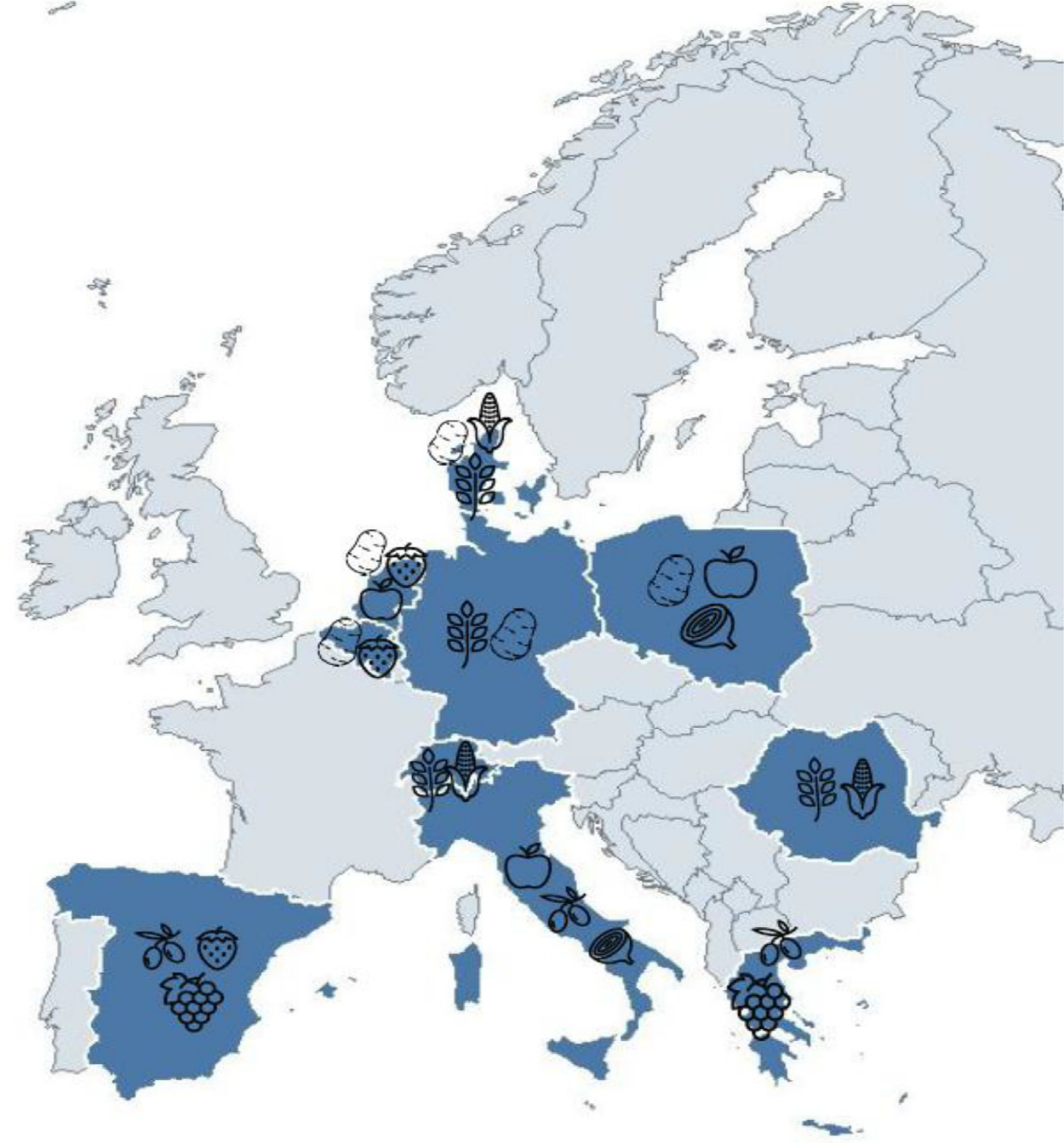
Geographic distribution of survey responses across 10 EU partner countries.

The data were collected as part of the Horizon Europe project SUPPORT (Supporting the Uptake of Integrated Pest Management and Low-Risk Pesticide Use)^3^. It is a transdisciplinary project with 25 National Crop Clusters in 10 different countries (see Table 1). The overall goal of the project is to pave the way towards more widespread IPM adoption enabling a fundamental reduction of pesticide use and risk in European agriculture to reach respective policy goals. The project aims to provide scientific knowledge to inform public policies and the private sector. The data collected in this context shall serve to identify barriers and opportunities of IPM adoption in different production systems in Europe. Special emphasis is on different factors shaping farmers’ decision-making processes in heterogeneous socio-ecological environments (Fig. 1).

**Table 1:** Overview of data collection in National Crop Clusters.

Country	Crop	Number of focus group participants	Data collection*	Fully completed questionnaires
Denmark	Wheat	09	NCC + Agency	48
	Maize	08		33
	Potatoes	08		28
Germany	Wheat	15	NCC + Agency	198
	Potatoes	15		214
Italy	Apples	23	NCC + Agency	127
	Olives	13		230
	Onions	16		125
Netherlands	Strawberries	08	NCC + Agency	30
	Apples	08		50
	Potatoes	08		61
Belgium	Strawberries	03	NCC	61
	Potatoes	02		194
Poland	Apples	17	NCC + Agency	158
	Potatoes	09		209
	Onions	12		148
Romania	Wheat	18	NCC + Agency	248
	Maize	18		203
Spain	Grapes	23	NCC + Agency	165
	Strawberries	30		99
	Olives	30		198
Switzerland	Wheat	05	NCC	800
	Maize	05		747
Greece	Grapes	NA	NCC	148
	Olives	14		162

⁎ Data collection periods differed across National Crop Clusters. The Swiss survey was conducted earlier (March-June) in 2024, the Greek survey on olive was conducted from March to July 2024, while the remaining National Crop Cluster surveys were administered over a longer period between March and December 2024.

## 3 Data Description

Data were collected on the adoption of IPM practices across 25 crop-country combinations, covering 10 countries and 8 crops grown in various production systems (both annual and perennial) for processing and fresh market use (see Supplementary Figs.: S2.2, S2.3). The survey included different sets of IPM practices for each National Crop Cluster. The dataset captures information on the current adoption levels of IPM practices, as well as the measures farmers would choose if required to reduce pesticide use by 50%. Additionally, it provides details on both farm and farmer characteristics (see Table 2), individual preferences and attitudes towards IPM practices, information sources, perceived barriers to IPM adoption, non-cognitive skills, and risk preferences. For confidentiality, personal identifiers such as names or email addresses, along with qualitative data like comments and feedback, were removed. The original questionnaire and codebooks for all 25 National Crop Clusters, detailing the specific IPM practices, variables, and descriptions, are available through the ETH Zürich Research Collection, including:

• Readme file (PDF) indicates the overview of the dataset contents and file structure.
• Codebook (CSV format) - description of all variables and their coding.
• Raw data file (CSV format) – anonymized farmer responses for the specific National Crop Cluster.
• The original questionnaire used (in National Crop Cluster specific language) in PDF format.
• The original questionnaire translated in English in PDF format.

**Table 2:** Overview of selected farm and farmer characteristics across countries and National Crop Clusters in the IPM large-scale survey in 2024.

	Belgium		Germany		Grece		Italy			Poland			Romania		Spain			Switzerland	
	Potato	strawberry	potato	wheat	Grape	olive	apple	olive	onion	apple	Onion	potato	maize	wheat	grape	olive	strawberry	wheat	maize
Age* (in years)	52.0	53.0	40.0	39.0	47.0	45.0	41.0	43.0	45.0	39.0	39.5	40.0	41.0	40.0	42.0	43.0	37.0	49.0	50.0
Female farmers (%)	4.6	8.2	23.8	16.2	11.5	19.8	22.1	22.6	32.0	31.0	31.8	31.1	25.1	20.2	14.6	23.2	36.4	5.0	6.9
Organic producers (%)	4.1	16.4	65.4	60.1	8.8	40.1	80.3	69.1	72.0	63.3	71.0	51.2	41.4	39.5	51.5	48.5	66.7	22.0	25.0
Farm size* (in ha)	45.0	12.0	80.0	75.0	4.0	5.8	50.0	10.0	25.0	10.0	26.0	15.0	87.5	100.0	35.0	30.0	18.5	29.5	28.8
Share of land under specific crop* (%)	12.5	12.5	63.0	50.5	63.0	88.0	63.0	63.0	38.0	63.0	38.0	38.0	38.0	38.0	63.0	88.0	63.0	22.1	21.8

Note: * For age, farm size, and share of land devoted to specific crops, median values are reported. Overall, the results presented in this table are based entirely on survey responses from participating farmers and may not fully represent all farmers of the respective crops at the national level; therefore, these overview statistics shall be interpreted with caution. For example, the proportion of organic producers or reported farm size may differ from national averages, reflecting the characteristics of survey respondents. This is partly due to the survey distribution strategy, which relied on the project’s national partners. Although organic farmers apply IPM measures [[15], [16], [17]], they may differ systematically from conventional producers in their pest management strategies, advisory networks, and attitudes toward pesticide reduction, this feature should be considered when interpreting variables related to current IPM uptake, perceived effectiveness, crop loss estimates, and information sources. In addition, the use of mixed data collection approaches may introduce self-selection bias, particularly in National Crop Clusters with smaller sample sizes. Although data were collected in the Netherlands and Denmark, national crop clusters from these countries are not included in this overview table due to limited observations and concerns regarding potential bias. All raw data underlying the tables and figures are freely available through the linked ETH Research Collection entries. To facilitate access, the repository structure follows a standardized NCC-specific format, with each entry containing the raw data file, codebook, questionnaire, and README documentation. The table has been created based on the outlier information provided by the National Crop Clusters. A complete list of reported outlier values is available in the Supplementary Materials (Table S1).

## 4 Experimental Design, Materials and Methods

To develop the core questions of our survey on IPM practices we first developed a list of practices based on the framework by Barzman et al. 2015 [1], which also reflects directive 2009/128/EC of the European Parliament to achieve the sustainable use of pesticides [2]. In addition, both overarching literature and National Crop Cluster-specific literature were used to identify IPM measures relevant for the different crops and production contexts. As a next step, to derive National Crop Clusters specific lists of IPM practices, in each National Crop Cluster focus groups were conducted in 2023 to discuss IPM practices that are currently used and could be applicable in the future with regional farmers, advisors, crop specialists and plant protection experts. Table 1 provides an overview of the number of participants who took part in the focus group discussion. For each candidate IPM measure, the National Crop Cluster used a structured fact sheet that was reviewed and validated by the focus group participants. The fact sheet included information on how the measure is used, when it is applied in the cropping cycle, which pest, disease, or weed problem it targets, and to which IPM pillar and principle it belongs. It also included information on whether the measure can be used in organic production, its efficacy, labour and investment costs, effects on yield and quality, potential to reduce pesticide use, and current level of implementation. In addition, participants could identify measures that had been missed during the preparation of the IPM lists. The eight principles of IPM were also discussed during the focus groups to ensure a common understanding among participants. This approach allowed us to maintain comparability across National Crop Clusters while accounting for crop- and country-specific differences. The fact-sheet template used in the focus-group process is provided in the Supplementary Material, S4. The online survey, available in nine languages (Danish, Dutch, French, German, Italian, Polish, Romanian, Spanish, and Greek), was distributed to farmers in the project’s National Crop Clusters between March and December 2024. Local project partners sent survey invitations via email, using email addresses from census databases (e.g., in Switzerland), connecting with farmer associations, and sharing the survey invitation in newsletters and on websites targeted at farmers. The project at large built-up national communities that were leveraged to share the surveys among farmers. Due to insufficient initial response numbers, the surveys were additionally distributed through a market research agency for some countries (see Table 1 for an overview). Specifically, Bilendi (a European panel-based market research agency specializing in online data collection) was hired to collect additional responses because it provided an opportunity to collect coherent farm-level data across different European countries. Bilendi used control questions to identify farms in the targeted population. The questionnaire was created and hosted on the online platform LimeSurvey. Prior to launch, the survey was pre-tested for clarity, wording, and user-friendliness with farmers at the Canton of Zurich's agricultural school in Switzerland. The survey did not follow a randomized sampling design and shall therefore might not be considered fully representative of the national farmer populations in each National Crop Cluster. Recruitment was coordinated by the national partners and depended on the availability of crop- and country-specific contact channels, including agency databases where available, farmer or producer associations, cooperatives, newsletters, magazines, social media, and sector-specific events. As a result, sample sizes and sample composition differ across National Crop Clusters. In several National Crop Clusters, the proportion of organic respondents is higher than national averages for the respective crop sectors, which likely reflects both the survey distribution channels and self-selection of respondents. This feature may affect responses in sections related to crop protection practices, crop loss perceptions, perceived effectiveness of IPM measures, and information sources.

The questionnaire (a sample questionnaire is provided in Supplementary, S3) was structured in the following sections:

• Information on the farm and the farmer
• Expected crop losses with and without plant protection strategies
• Current adoption of IPM measures
• Effective measures to reduce pesticide use and risk by 50%
• Attitudes towards pesticide reduction goals and information sources
• Personal perceptions and preferences
  1. Information on farm and farmer
    Following initial screening questions on crop production, decision-making responsibilities, and main production type, this section focused on farm structure. Questions covered farm size (in hectares), number of employees, postal code, proportion of leased land, percentage of land dedicated to the specific crop production, estimated yield potential, organic certification, label production, and participation in agri-environmental schemes. Additional questions addressed farmer characteristics, including highest education level, birth year, gender, farm succession planning, and crop insurance use, following Knapp et al. 2021 [3].
  2. Crop Losses with and without Plant Protection Strategies
    The survey contains questions on perceived baseline impacts of different pest sources on crop revenue and the effectiveness of applied control measures. So, we first asked the farmer to consider the anticipated crop revenue losses (average revenue loss percentage) due to fungi, insects, and weeds under a hypothetical scenario where no plant protection measures were in place. We then subsequently asked the farmers to provide their estimates of the actual crop revenue losses caused by these same factors with their current plant protection strategy applied.
  3. Adoption of IPM Measures
    In this part of the questionnaire, farmers were asked about their current use of IPM measures. The list of IPM measures was structured based on the eight IPM principles by Barzman et al. 2015 [1]. The survey and dataset contained farmers’ indications for each IPM measure separately. In addition, the questionnaire provided a further categorization of these eight principles into three major parts to simplify follow up analysis: i) preventive strategies, ii) intervention measures, and iii) evaluation practices. First, preventive strategies largely include early designs and actions undertaken during the cropping system phase to reduce the presence of pests. These strategies also include regular monitoring of harmful pests and following local warnings to inform intervention decisions. Second, intervention measures consist of different sequences of control alternatives that can start with non-chemical methods, for example. Finally, the evaluation techniques indicate the assessment criteria that farmers might consider improving the pest management process [1]. Each National Crop Clusters got a unique list of preventive measures tailored to the specific crop and production system (see details above), while the intervention and evaluation measures applied universally across National Crop Clusters. Farmers were also asked to report the current number of pesticide applications per season, including herbicides, insecticides, acaricides, fungicides, and others.
  4. Effective IPM Measures for a 50% Reduction in Pesticide Use
    Here, farmers were asked to imagine a scenario where they must reduce pesticide use by 50%, following the goals postulated in the EU’s Farm to Fork Strategy and Switzerland’s national action plan on pesticides [4,5]. They are prompted to select a combination of IPM measures to meet this goal, either by building on their existing strategies from the previous question or by choosing an entirely new set of practices. We then asked the growers to rate the effectiveness of the IPM measures they have selected to control plant diseases, insects/mites, and weeds in the respective crop compared to a scenario without any crop protection. The response options ranged from 0 (not effective at all) to 10 (very effective), with an additional option “I do not know”.
  5. Enablers and Barriers to Adopting IPM Measures
    This section begins by assessing the farmer’s willingness to adopt the previously selected measures for achieving a 50% reduction in pesticide use [6,7]. Farmers can choose from eight statements representing different levels of willingness, ranging from ``I do not plan to implement the selected measures because there is no reason to do so'' to ``I have already implemented these measures and will adopt more of them in the coming years.” Following this, the survey explores potential barriers the farmer might face in transitioning to the 50% pesticide reduction strategy [8,9]. Respondents rate the significance of various obstacles, such as lack of knowledge or experience and perceived low effectiveness of alternative measures, using a Likert scale ranging from 0 to 10 to indicate the degree to which each factor may hinder adoption, including the option ‘I do not know’.
  6. Farmers’ attitudes towards pesticide reduction goals and information sources
    In this section, farmers are asked to evaluate several statements about the potential effects of a 50% reduction in pesticide use, considering aspects like the impact on farmers’ health, public health, and the environment. Farmers will rate each statement on a Likert scale ranging from -5 (Very Negative) to +5 (Very Positive) with the response alternative ‘I do not know.
    Additionally, the section includes a question about the importance of various factors in farmers' crop protection decisions, such as subsidies for sustainable crop protection methods, achieving high yields, maintaining biodiversity, and others [10,11]. Respondents will rate the importance of these factors on a Likert scale from 0 (Not Important at All) to 10 (Very Important). Farmers are also asked to indicate the sources they rely on for information about plant protection, such as extension services, newspapers, social media, and other farmers [12]. In a follow-up question, farmers indicated which of these information sources they trust the most.
  7. Personal perceptions and preferences
    This section of the questionnaire examines farmers' attitudes and perceptions regarding crop protection. Specifically, it asks farmers to indicate their level of agreement with statements related to innovativeness, self-efficacy, and locus of control [3,13]. Additionally, questions assess time preferences [14] and risk tolerance. Willingness to take risks is measured on a 0 to 10-point Likert scale across various domains, including crop production, market and pricing, plant protection, and agriculture in general [3,8].

### 4.1 Data cleaning and preparation

Before uploading the datasets to the repository platform, basic cleaning steps were applied to the survey data which includes removing duplicates and incomplete responses, merging responses originating from both agency contacts and National Crop Clusters contacts. The Swiss survey data have been matched with structural farm characteristics from census data provided by the Federal Office for Agriculture, specifically agricultural area, number of employees, location, percentage of wheat or maize on arable land, and year of birth of farmer. Sensitive details such as farm location (for example ZIP code), and email addresses were excluded to minimize the risk of traceability of the participants. Variable names were harmonized across National Crop Clusters to ensure a common structure. Apart from these steps, the data remain in raw form. Therefore, researchers can apply further plausibility checks according to their specific analytical needs. For example, we also provide information on outlier identification provided by the National Crop Clusters in Supplementary Table S1.

## Limitations

Due to challenges in the data collection process across several National Crop Clusters, the entire data collection period has been extended and may span beyond a single cropping season (from March to December 2024). There is a potential sampling bias in our dataset. For example, organic farmers are overrepresented compared to their proportion in the national farming population. One possible reason for this is that organic farmers may be more likely to respond to surveys or be more motivated to participate due to their interest in sustainable crop protection practices, extension/advisory services, etc. Another possible reason is that a considerable percentage of farmers are in the process of transitioning to organic farming (e.g., olive farmers in Greece). In addition, the use of mixed data collection approaches (e.g., online surveys, farmer associations, cooperative websites, social media, and events) may introduce self-selection biases. Finally, we collected a large sample (N = 4,684), but we couldn't ensure that it was fully representative of every country and every crop cluster (e.g., some regions or farm sizes may have been under-sampled, see Supplementary Fig. S2.1 for a graphical overview).

## Ethics Statement

This study and data collection was approved by the ETH Zurich Ethics Commission (reference number: EK 2023-N-318). Informed consent was obtained from respondents with an online form in the beginning of the survey (a copy of the consent form is included with the original questionnaire on the ETH research collection).

## Credit Author Statement

**Akter, Sharmin:** Conceptualization, Methodology, Data collection and curation, Visualization, Writing – original draft, Writing – review & editing. **Kreft, Cordelia:** Conceptualization, Methodology, Data collection and curation, Writing – original draft, Writing – review & editing. **McCallum, Chloe**: Conceptualization, Methodology, Data collection, Writing – review & editing. **Möhring, Niklas**: Conceptualization, Methodology, Writing – review & editing. **Spirig, Andri:** Data curation, Visualization, Writing – review & editing. **Erwin Wauters:** Methodology, Resources, Data collection, Writing – review & editing. **Clara Sciffer:** Methodology, Resources, Data collection, Writing – review & editing. **Vasiliki Inglezou:** Methodology, Resources, Data collection, Writing – review & editing. **Georgios Dimitroulas:** Methodology, Resources, Data collection, Writing – review & editing. **Silke Dachbrodt-Saaydeh:** Methodology, Resources, Data collection, Writing – review & editing. **Lars Ole Hingst:** Methodology, Resources, Data collection, Writing – review & editing. **Maria Toader:** Methodology, Resources, Data collection, Writing – review & editing. **Cristina Radu:** Methodology, Resources, Data collection, Writing – review & editing. **Juan Sagarna:** Methodology, Resources, Data collection, Writing – review & editing. **Patricia de Almandoz:** Methodology, Resources, Data collection, Writing – review & editing. **Joanna Golian:** Methodology, Resources, Data collection, Writing – review & editing. **Joanna Pulawska:** Methodology, Resources, Data collection, Writing – review & editing. **Dominika Boguszewska-Mańkowska:** Methodology, Resources, Data collection, Writing – review & editing. **Milena Pietraszko:** Methodology, Resources, Data collection, Writing – review & editing. **Dorota Labanowska-Bury:** Methodology, Resources, Data collection, Writing – review & editing. **Hanka Sobczyńska:** Methodology, Resources, Data collection, Writing – review & editing. **Alberto Sturla:** Methodology, Resources, Data collection, Writing – review & editing. **Laura Viganò:** Methodology, Resources, Data collection, Writing – review & editing. **Maria Grazia Tommasini:** Methodology, Resources, Data collection, Writing – review & editing. **Mattia Dall’Ara:** Methodology, Resources, Data collection, Writing – review & editing. **Carolina Gattorno:** Methodology, Resources, Data collection, Writing – review & editing. **Frans Geijs:** Methodology, Resources, Data collection, Writing – review & editing. **Willem Hendriks:** Methodology, Resources, Data collection, Writing – review & editing. **Yaite Cuesta Arenas:** Methodology, Resources, Data collection, Writing – review & editing. **Harrie Pijnenburgh:** Methodology, Resources, Data collection, Writing – review & editing. **Sophie Louise Appel:** Methodology, Resources, Data collection, Writing – review & editing. **Jens Erik Jensen:** Methodology, Resources, Data collection, Writing – review & editing. **Erato Lazarou:** Methodology, Resources, Data collection, Writing – review & editing. **Stella Bitsika:** Methodology, Resources, Data collection, Writing – review & editing. **Finger, Robert:** Conceptualization, Methodology, Data collection, Writing – original draft, Writing- review & editing, Project administration and Supervision

## Data Availability

ETH research collectionData collection on the uptake of integrated pest management (IPM) practices in European agriculture (Original data)

## Appendix Supplementary materials

Image, application 1

## Footnotes

3 Further information on the SUPPORT project is to be found on the project website: HE Support (he-support.eu).

## References

[1] Barzman (2015). Eight principles of integrated pest management. Gron. Sustain. Dev..

[2] https://eur-lex.europa.eu/eli/dir/2009/128/2009-11-25

[3] Knapp (2021). Preferences, personality, aspirations, and farmer behavior. Agric. Econ..

[4] Finger (2021). No pesticide-free Switzerland. Nat. Plants.

[5] Schneider (2023). Pesticide reduction amidst food and feed security concerns in Europe. Nat. Food.

[6] Bamberg (2013). Changing environmentally harmful behaviors: a stage model of self-regulated behavioral change. J. Env. Psychol..

[7] Doran (2022). Understanding farmers’ conservation behavior over time: A longitudinal application of the transtheoretical model of behavior change. J. Env. Manag..

[8] Möhring & Finger, 2022: Pesticide-free but not organic: Adoption of a large-scale wheat production standard in Switzerland, 10.1016/j.foodpol.2021.102188 PMC8803942 35128011

[9] Möhring & Finger (2022). Data on the adoption of pesticide-free wheat production in Switzerland. Data Br..

[10] Pedersen (2012). Optimising the effect of policy instruments: a study of farmers’ decision rationales and how they match the incentives in Danish pesticide policy. J. Environ. Plan. Manag..

[11] Lindenberg & Steg (2007). Normative, gain and hedonic goal frames guiding environmental behavior. J. Soc. Issues.

[12] Wuepper (2021). Does it matter who advises farmers? Pest management choices with public and private extension. Food Policy.

[13] Kreft (2021). The role of non-cognitive skills in farmers’ adoption of climate change mitigation measures. Ecol. Econ..

[14] Falk (2018). Global evidence on economic preferences. Q. J. Econ..

[15] Crowder (2010). Organic agriculture promotes evenness and natural pest control. Nature.

[16] Lotter (2003). Organic agriculture. J. Sustain. Agric..

[17] Finger (2024). Towards sustainable crop protection in agriculture: a framework for research and policy. Agric. Syst..

