# Supplementary material for: Data on the uptake of Integrated Pest Management (IPM) practices in European agriculture
Source: Data Brief. 2026 Jul 13;67:113075. doi: 10.1016/j.dib.2026.113075 (PMC13396935; doi:10.1016/j.dib.2026.113075)
Supplement: Supplementary file 1 [file mmc1.docx]

Supplementary Materials

Table S1. Outlier information reported by National Crop Clusters for open-ended survey questions

|  | Belgium | | Germany | | Grece | | Italy | | | Poland | | |
| --- | --- | --- | --- | --- | --- | --- | --- | --- | --- | --- | --- | --- |
|  | potato | strawberry | potato | wheat | grape | olive | apple | olive | onion | apple | onion | potato |
| Farm size (ha) | NA | Remove ID = 167 | Remove ID = 99 | NA | 10 | NA | 120 | 150 | 100 | 80 | 200 | 200 |
| Treatment freq. (per growing season) | Herbicides: 10  Fungicides: 5-20  Insecticides/Acaricides: 12 | Herbicides: <10  Fungicides: 25  Insecticides/Acaricides: <20 | NA | NA | 13-15 (not distinguished) | NA | NA | NA | NA | 40 (not distinguished + including fertilization and bio simulants) | NA | 9 (not distinguished) |
| Labour (full time employees) | NA | <10 | NA | NA | 5 | NA | NA | NA | NA | NA | NA | 10 |
| Insurance premium (€) | NA | NA | 30-100 per ha | 30-100 per ha | 0.5-0.75 per kg | Oil olives: 48.70 per ha  Table olives: 150 per ha | 3500 per ha | 200 per ha | 650 per ha | 9% of expected yield | Social security: 18.47 per month  Pension contribution: 39.07 per month  Crop insurance: 402.54- 426.22 per month | NA |

Table S1: Continued…

|  | Romania | | Spain | | | Switzerland | | Notes |
| --- | --- | --- | --- | --- | --- | --- | --- | --- |
|  | maize | wheat | grape | olive | strawberry | wheat | maize | For Switzerland: No outliers |
| Farm size (ha) | 50000 | 50000 | 1000 | 1000 | 50 | NA | NA |  |
| Treatment freq. (per growing season) | 10 (not distinguished) | 10 (not distinguished) | 25 (not distinguished) | 15 (not distinguished) | 35 (not distinguished) | NA | NA |  |
| Labour (full time employees) | 12.1 | 12.1 | 30 | 17 | 10 | NA | NA |  |
| Insurance premium (€) | 22 | 27.89 | 1000 per ha | 1000 per ha | 100 per ha | NA | NA | For Poland: PLN-euro exchange rate 0.24. 19 Jan, 10:21 UTC  For Romania: lei-euro exchange rate 0.20, 19. Jan., 12:28 UTC |

S2: Additional figures for the National Crop Clusters in the IPM large-scale survey in 2024


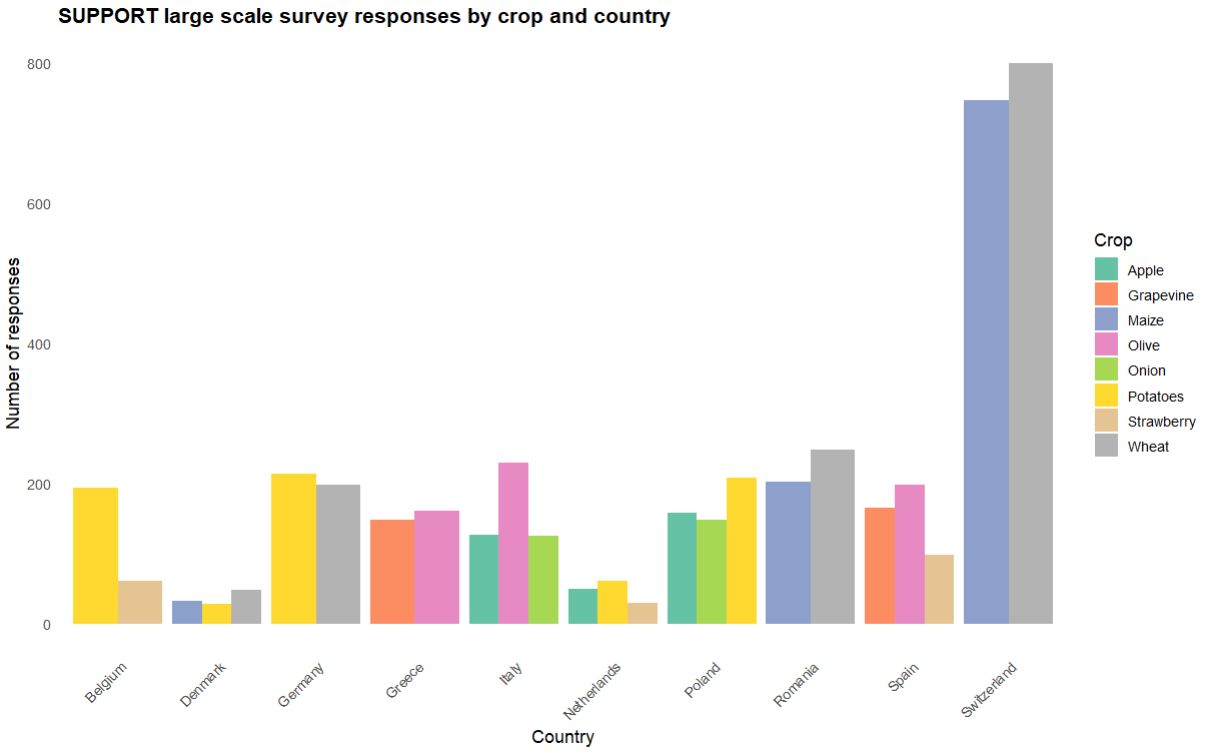


Figure S2.1: Distribution of survey responses across crops and participating countries.


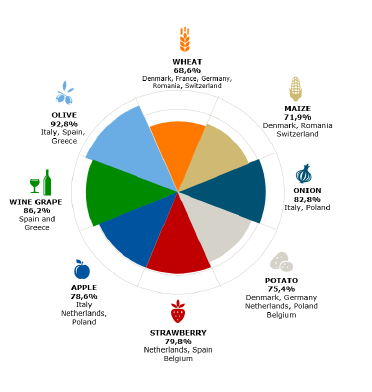


Figure S2.2: The European coverage of SUPPORT crop areas in the member states participating in our large-scale survey 2024.


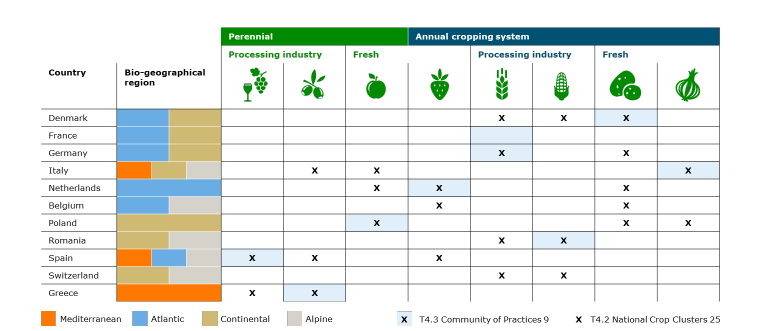


Figure S2.3: The 25 SUPPORT NCCs represent a high variety of crop systems both in perennial and in annual crops in Europe in the different bio-geographical regions*.*

S3: Sample questionnaire for olive producers in Greece. Other questionnaires are available in the ETH Research Collection. All questionnaires follow a common structure, with some questions tailored to specific countries and crops. For example, questions on production labels under which products are sold (Question 8), agri-environmental schemes (Question 9), education system (Question 10), IPM measures (Question 19), and National Crop Cluster–specific pest management information sources (Question 26).

**Dear olive producers**,

We are pleased that you are taking part in this survey on your current and future crop protection practices and perceptions. The survey will provide important insights into agricultural practice, advice and research.

The aim of the study is to find out which factors influence the decision of farmers’ plant protection strategies and the role of challenges and opportunities for the adoption of sustainable pest management in olive cultivation.

If you are interested, you will receive feedback on the results of the survey. Answering the survey only takes about **20 minutes**.

**Thank you in advance for your participation**.

Yours’ sincerely,

Cordelia Kreft (ETH Zürich) and NCC partner

If you have any further questions, please contact:

Sharmin Akter (sakter@ethz.ch)

Agricultural Economics and Policy Group, ETH Zurich

Thank you for your participation.

Consent

**Privacy policy label text:**

I hereby confirm that my participation is voluntary and consent that answers from this survey are used anonymously for scientific purposes under the data protection conditions given below.

**Privacy Policy Message:** Please read the text below carefully and get in contact if any questions remain.

**Contact people.**

Contact person (full name):                  Robert Finger (rofinger@ethz.ch)

                              Agricultural Economics and Policy Group, ETH Zurich

Conducting person:

                                                             Sharmin Akter (sakter@ethz.ch)

                                     Agricultural Economics and Policy Group, ETH Zurich

**Information about the study**

**What is the purpose of this study?**

The objective of the study is to collect information on integrated pest management (IPM) practices. We aim to identify factors facilitating or hindering the uptake of IPM measures, e.g., concerning heterogeneous farm structures and environmental conditions as well as individual behavioral characteristics. We also aim to elicit the future developments of farmers IPM uptake. Survey results will only be used to provide feedback on the potential and future developments IPM uptake to farmers and stakeholders. Results can also improve policy design.

**Who can participate?**

The survey is aimed at Olive producers in Greece.

**How much time will I be expected to invest?**

The survey will take about 20 minutes.

**Are there any risks or benefits?**

There are no risks involved. Data will be treated anonymously, and no sensitive information is shared with third parties. At the end of the project, farmers (if requested) will receive a report with a summary of the results.

**What are my rights to the data?**

You have the right to request information about the processing of your personal data at any time. You can also ask for incorrect, inaccurate or incomplete personal data to be corrected, barred for processing, or erased when it’s no longer needed or if processing it is unlawful. You may revoke your consent to the processing of your personal data at any time without giving reasons. Your contact person for related requests is given above.

**Which data is collected?**

Data on your personal assessments, perceptions, preferences towards sustainable agricultural practices, farm information as well as your evaluation of key constraints and drivers on Integrated Pest Management practices.

**How is my data treated?**

The data will be processed and stored anonymously only at servers of ETH Zürich, in Switzerland. No conclusions can be drawn about individual participants. Only people involved in the project can access the original data. The data is anonymized before it is archived and made accessible for research purposes. The members of the ETH Zurich ethics committee can view the original data for testing and control purposes, but with strict observance of confidentiality.

**Who funds the study?**

The study is financed by the programme HORIZON Europe.

**How am I insured?**

Adverse health effects that are directly caused by the study and can be demonstrated to be attributable to fault on the part of the project team or ETH Zurich are covered by ETH's liability insurance (Basler Versicherungen, policy no. 30/4.078.362). You are responsible for insuring yourself against any other adverse health effects such as might occur, for instance, in connection with the trip to or from the place where the study is conducted.

**Who examined the study?**

This study was approved by the ETH Zurich Ethics Commission as proposal EK 2023-N-318.

**Complaints office**

The secretariat of the ETH Zurich Ethics Committee is available to help you with complaints in connection with your participation. Contact: *ethics@sl.ethz.ch* or 0041 44 632 85 72.

I, the participant, confirm hereby that:

- I have read and understood the study information. My questions have been answered completely and to my satisfaction.
- I comply with the inclusion and exclusion criteria for participation described above. I am aware of the requirements and restrictions to be observed during the study.
- I have had enough time to decide about my participation.
- I participate in this study voluntarily and consent that my personal data be used as described above.

I understand that I can stop participating at any moment.

**Feedback**

- Would you like to receive the results of the survey? We will send you an overview of the results.
- Yes
- No

**Selection control question**

- Do you produce olives on your farm?
- Yes
- No
- Are you involved in making the decisions about olive production on your farm?
- Yes
- No
- Which of the following best describes your production?
- Only olives
- Olive seeds
- Olives and olive seeds

Section 1: Information about the farm and farm manager

The following section contains questions on the characteristics of your farm and olive production as well as some information about you.

1. What is your total farmland in hectare? _____________________________________________

2. How many full-time labour equivalent employees does your farm have (including yourself and family members)? _____________________________________________________________

3: To anonymously link your answers with weather data from your region, please fill in your zip code: _________________________________________________________________________

4: Which share of your arable land are you leasing?

- - 0-25%
  - 26-50%
  - 51-75%
  - 76-100%

5: Which share of your arable land is devoted to olive production?

- - 0-25%
  - 26-50%
  - 51-75%
  - 76-100%

6: Which share of your total household income comes from the following production branch of your farm (including direct payments)?

Please indicate the approximate income share for each branch. If you have no income from that branch, please put 0.

|  |  |
| --- | --- |
| Olive production |  |
| Arable farming (including olive production) |  |
| Other agricultural production on the farm |  |
| Other on-farm activities (e.g., agrotourism) |  |
| Off-farm sources |  |

7: How do you estimate the production potential of your land compared to the rest of Greek olive production?

| The worst in Greece 1 | 2 | 3 | 4 | 5 | 6 | 7 | 8 | 9 | The best in Greece  10 |
| --- | --- | --- | --- | --- | --- | --- | --- | --- | --- |
| ☐ | ☐ | ☐ | ☐ | ☐ | ☐ | ☐ | ☐ | ☐ | ☐ |

8a: Do you produce olive under organic production?

- 1. Yes
  2. No

8b: Do you sell the majority of your olive under one of the following programs or labels? [More than one answer is possible].

- - Dimitra
  - AGRO 2.1
  - EU organic farming logo
  - Greece Agriculture
  - Naturland
  - None
  - Other

9: What direct payment programs do you participate in for olive?

- - Program of Rural Development
  - Integrated Management System in agricultural production
  - National Action Plan for the Rational Use of Agricultural Chemicals
  - Other
  - None

10: What is the highest education that you have completed?

- - Secondary Education/ Agricultural School
  - Higher Vocational Training Institute/Technological Educational Foundation
  - Agricultural University
  - Other……………..

11: Please indicate your year of birth____________________

12: What is your gender?

- - Female
  - Male
  - Rather not say

13: Has your farm succession already been arranged?

- - Yes
  - No
  - Not yet relevant

14: Do you have crop yield insurance for olive production?

- - Yes
  - No

15: If “Yes”, how much do you approximately pay for the premium (€/ha)?

**Section 2: Crop losses**

| 16.Imagine you **do not use any crop protection**, what is the **average loss** of crop revenue (in %) you would expect from the given sources? |  |
| --- | --- |
| Plant diseases | % |
| Insects and mites | % |
| Weeds | % |
| 17. Now we go back to your **current crop protection strategy**. Given the strategy that you use, what is the **average loss** of crop revenue (in %) you would expect from the given sources? |  |
| Plant diseases | % |
| Insects and mites | % |
| Weeds | % |

18. What is the current average level (**number of treatments per cropping season**) of the following pesticide types in your olive production?

|  |  |
| --- | --- |
| Herbicides |  |
| Insecticides and Acaricides |  |
| Fungicides |  |
| Other |  |

**Section 3: Current IPM Measures**

For all questions on this page, please indicate whether you currently adopt the following pest management measures:

19a. To **prevent, monitor and/or control** the occurrence of **pests and weeds** in olive cultivation, I..

| practice field hygiene (e.g., certified seeds) |
| --- |
| create and promote ecological habitats (e.g., flower strips, hedges or wildflowers to support beneficial insects) |
| use cover crops |
| monitor and scout plants to reduce stress (e.g. pruning and optimal nutrition and irrigation) |
| use biological control (i.e. beneficial insects such as predatory mites, lady bugs, lacewings and parasitic wasps) |
| use sexual confusion techniques (e.g. pheromone traps) |
| choose varieties with high resistance (e.g., varieties resistant to fungi/insects/other pests) |
| check the pest and weed infestation at regular intervals (e.g. visual inspections, traps or nets) |
| use the prediction and warning systems for pests and diseases that are offered (e.g., by crop protection services) |
| base my decision on official (damage) thresholds |
| do not implement any of the measures mentioned. |

19b. When **using pesticides** in olive production, I...

| prefer biological products |
| --- |
| reduce the frequency of application |
| apply them only to the (partial) areas where they are needed with hoe band sprayers |
| use lower spray pressure, adjusted water application rate, reducing driving speed, etc. |
| use pesticides that work differently or have different resistance classes |
| clean the spray equipment only in the areas provided for this purpose |
| do not use any pesticides in olive production |

19c. To **assess the success** of my crop protection strategy in olive production, I ...

| check the pest infestation before and after the implemented measure |
| --- |
| test the effectiveness of the measure by leaving a small area of the field untreated and comparing it with the treated area |
| document the results |
| don't systematically evaluate my crop protection strategy |

**Section 4: Effective IPM measures to reduce 50% of current pesticide use**

**Imagine** you have to **reduce your current pesticide use and the associated risk by 50%**. Which **combination of the following measures would you implement** to reach this goal?

Note that you can choose your existing crop protection strategy (which has been pre-selected in the question below) and add more measures – or choose a completely new set of measures.

20a. To **prevent, monitor and/or control** the occurrence of **pests and weeds** in olive cultivation, I would..

| practice field hygiene (e.g., certified seeds) |
| --- |
| create and promote ecological habitats (e.g., flower strips, hedges or wildflowers to support beneficial insects) |
| use cover crops |
| monitor and scout plants to reduce stress (e.g. pruning and optimal nutrition and irrigation) |
| biological Control (i.e. beneficial insects such as predatory mites, lady bugs, lacewings and parasitic wasps) |
| use sexual confusion techniques (e.g. pheromone traps) |
| choose varieties with high resistance (e.g., varieties resistant to fungi/insects/other pests) |
| check the pest and weed infestation at regular intervals (e.g. visual inspections, traps or nets) |
| use the prediction and warning systems for pests and diseases that are offered (e.g., by crop protection services) |
| base my decision on official (damage) thresholds |
| do not apply any of these measures |

20b. When **using pesticides** in olive production, I would..

| prefer biological products |
| --- |
| reduce the frequency of application |
| apply them only to the (partial) areas where they are needed with hoe band sprayers |
| use lower spray pressure, adjusted water application rate, reducing driving speed, etc. |
| use pesticides that work differently or have different resistance classes |
| clean the spray equipment only in the areas provided for this purpose |
| not use any pesticides in olive production |

20c. To **assess the success** of my crop protection strategy in olive production, I would..

| check the pest infestation before and after the implemented measure |
| --- |
| test the effectiveness of the measure by leaving a small area of the field untreated and comparing it with the treated area |
| document the results |
| don't systematically evaluate my crop protection strategy |

20d. How effective do you think each measure is to control plant diseases, insects/mites and weeds in olive compared to a scenario without any crop protection?

Note: the measures shown here are measures you currently use or selected to use to meet a 50% reduction in pesticide use and risks.

| **Measure** | **How effective do you think the measure is to control plant diseases, insects/mites and weeds in olive compared to a scenario without any crop protection?** | | | | | | | | | |  |
| --- | --- | --- | --- | --- | --- | --- | --- | --- | --- | --- | --- |
| List of IPM measures | Not effective at all  0 |  |  |  |  |  |  |  |  | Very effective  10 | I don’t know |
| [[here previous measures selected are shown]] | ☐ | ☐ | ☐ | ☐ | ☐ | ☐ | ☐ | ☐ | ☐ | ☐ | ☐ |
|  |  |  |  |  |  |  |  |  |  |  |  |
|  |  |  |  |  |  |  |  |  |  |  |  |

**Section 5: Intention to adopt IPM measures and barriers**

21.You chose the below combination of measures to reduce your current pesticide level by 50%. (shown by a table)

22. Which of the following statements best describes your intention to adopt the combination of measures shown above in the **next 3-5 years**?

|  |  |
| --- | --- |
| I am not planning to adopt them because I see no reason why I should do it. |  |
| I am not planning to adopt them, because it is technically impossible for me to do so |  |
| I am not planning to adopt them because it is not profitable for my farm. |  |
| I would like to adopt them, but I am not sure about how I should technically do so | ☐ |
| I would like to adopt them, but I am not sure about how I should economically do so | ☐ |
| I will adopt them, but I have not put it into practice yet |  |
| I have adopted them and will maintain this for coming years |  |
| I have adopted them and will adopt more measures in the coming years |  |

23. What are the main barriers for you for switching from your current strategy to the 50% reduction strategy you have chosen?  Please rate the following factors according to how relevant you perceive them.

| **Perceived Barrier** | **How important do you perceive this factor in hindering you from taking up measures to reduce pesticide use and risk?** |
| --- | --- |
| Lack of knowledge/experiences/insufficient training and technical support about alternative pest control | 0 (not at all important) to 10 (very important)/ I do not know |
| No availability of substitutes for pesticides |  |
| Low effectiveness of substitutes for pesticides |  |
| High costs of substitutes for pesticides |  |
| Lack of extension service on IPM |  |
| Weed pressure in olive |  |
| Weed pressure in overall rotation |  |
| Machinery for mechanical weed not available |  |
| High additional workload |  |
| Incompatible soil conditions for mechanical weeding |  |
| Lack of social acceptance |  |
| Yield decrease |  |
| Quality decrease |  |
| Volatility of yield |  |
| Volatility of quality |  |
| No additional price premium |  |
| Supply-chain problems (no possibility to transport, store and market low-pesticide production separately) |  |
| Please list any other barriers that hinder you switching from your current to the 50% reduction strategy you have chosen? |  |

**Section 6: Assessment of 50% pesticide reduction and Information source of IPM measures**

24. How would you assess the following statements about the wider effects of a 50%-reduction of pesticides in overall olive production? (-5 very negative to +5 very positive)

|  | -5  Very negative | -4 | -3 | -2 | -1 | 0 | +1 | +2 | +3 | +4 | +5  Very positive |
| --- | --- | --- | --- | --- | --- | --- | --- | --- | --- | --- | --- |
| Health of farmers will be | ☐ | ☐ | ☐ | ☐ | ☐ | ☐ | ☐ | ☐ | ☐ | ☐ | ☐ |
| Health of general public will be |  |  |  |  |  |  |  |  |  |  |  |
| The nature and environment will be |  |  |  |  |  |  |  |  |  |  |  |
| Olive yields in Greece per hectare will be |  |  |  |  |  |  |  |  |  |  |  |
| Soil fertility will be |  |  |  |  |  |  |  |  |  |  |  |
| Consumers’ willingness to pay higher prices will be |  |  |  |  |  |  |  |  |  |  |  |
| Long-term sustainability and resilience of olive production will be |  |  |  |  |  |  |  |  |  |  |  |
| Workload on my farm and others will be |  |  |  |  |  |  |  |  |  |  |  |
| Working conditions in olive production will be |  |  |  |  |  |  |  |  |  |  |  |

25. How important are the following factors for your decisions regarding crop protection in olive production generally?

|  | Not important at all  0 |  |  |  |  |  |  |  |  | Very important  10 | |
| --- | --- | --- | --- | --- | --- | --- | --- | --- | --- | --- | --- |
| Subsidies for implementing sustainable crop protection methods. | ☐ | ☐ | ☐ | ☐ | ☐ | ☐ | ☐ | ☐ | ☐ | ☐ | ☐ |
| Achieving a high olive yield (dt/ha). |  |  |  |  |  |  |  |  |  |  |  |
| Having “clean” fields (few weeds visible). |  |  |  |  |  |  |  |  |  |  |  |
| Keeping a high biodiversity on my fields. |  |  |  |  |  |  |  |  |  |  |  |
| Achieving a high income from olive production (incl. direct payments). |  |  |  |  |  |  |  |  |  |  |  |
| Reduction of my workload related to olive production. |  |  |  |  |  |  |  |  |  |  |  |
| Consumers’ demand for products produced with reduced pesticide use. |  |  |  |  |  |  |  |  |  |  |  |
| Contribution to a healthy environment. |  |  |  |  |  |  |  |  |  |  |  |
| Long-term increase of soil fertility on my agricultural land. |  |  |  |  |  |  |  |  |  |  |  |
| Other farmers’ crop protection methods |  |  |  |  |  |  |  |  |  |  |  |
| Social recognition. |  |  |  |  |  |  |  |  |  |  |  |
| Continuing family tradition. |  |  |  |  |  |  |  |  |  |  |  |

26 a. Where do you search for plant protection information?

- - - ELGO “DIMITRA”
    - National Union of Agricultural Cooperatives - ETHEAS
    - Panhellenic Confederation of Associations of Agricultural Cooperatives - PASEGES
    - Institute of Agricultural Economics and Sociology - INAGROK
    - Institute of Agricultural and Cooperative Economics - INASO
    - NATIONAL AGRICULTURAL NETWORK - EAD
    - Private consultants
    - Plant protection firms
    - Input suppliers
    - Other farmers
    - Farmer association
    - Producer organisations
    - Internet
    - Social media
    - Newspapers and professional journals
  1. *Other:* _____________________
  2. *I do not look for information on IPM*

26b. What is your most trusted and important source for information and advice for your pest management decisions?

Section 7: Perceptions and preferences of farmers

27. In this section, we would like to learn more about your **attitudes and perceptions** in the general context of **crop protection**. There are no right or wrong answers. It is all about your very personal assessments.

|  | I do not agree at all  0 |  |  |  |  |  |  |  |  | I fully agree  10 |
| --- | --- | --- | --- | --- | --- | --- | --- | --- | --- | --- |
| I am ready to implement innovative crop protection methods earlier than other farmers in the region. | ☐ | ☐ | ☐ | ☐ | ☐ | ☐ | ☐ | ☐ | ☐ | ☐ |
| I am open to new forms of crop protection, but I want to think through all aspects first. While doing so, I focus on the experiences of other farmers. |  |  |  |  |  |  |  |  |  |  |
| In principle, I only implement new crop protection measures if they have already been implemented by  others for a while and have proven themselves. |  |  |  |  |  |  |  |  |  |  |
| I rely on the tried and tested. Implementing new crop protection methods in my olive production is economically  too risky for me. |  |  |  |  |  |  |  |  |  |  |

28. How much do you agree with the following statements?

|  | I do not agree at all.  0 |  |  |  |  |  |  |  |  | I fully agree  10 |
| --- | --- | --- | --- | --- | --- | --- | --- | --- | --- | --- |
| Success in crop protection mainly depends on the abilities of the farmer. | ☐ | ☐ | ☐ | ☐ | ☐ | ☐ | ☐ | ☐ | ☐ | ☐ |
| Weed and pest infestation in olive is very hard to control for farmers. |  |  |  |  |  |  |  |  |  |  |
| Success in crop protection can hardly be influenced by the farmer. |  |  |  |  |  |  |  |  |  |  |
| If there is a problem in olive production, I usually find a solution. |  |  |  |  |  |  |  |  |  |  |
| I can solve most problems in crop protection if I make an effort. |  |  |  |  |  |  |  |  |  |  |
| I am confident that I can reduce pesticides and at the same time produce olive successfully. |  |  |  |  |  |  |  |  |  |  |

29. How willing are you to give up income that is beneficial for you/ the farm today in order to benefit more from it in the future?

| Not willing  0 |  |  |  |  |  |  |  |  | Very willing  10 |
| --- | --- | --- | --- | --- | --- | --- | --- | --- | --- |
| ☐ | ☐ | ☐ | ☐ | ☐ | ☐ | ☐ | ☐ | ☐ | ☐ |

30. How willing are you to take risks in the areas listed below?

|  | Not willing at all  0 |  |  |  |  |  |  |  |  |  | Very willing 10 |
| --- | --- | --- | --- | --- | --- | --- | --- | --- | --- | --- | --- |
| Olive production | ☐ | ☐ | ☐ | ☐ | ☐ | ☐ | ☐ | ☐ | ☐ | ☐ | ☐ |
| Market and Prices |  |  |  |  |  |  |  |  |  |  |  |
| Plant protection |  |  |  |  |  |  |  |  |  |  |  |
| Agriculture in general |  |  |  |  |  |  |  |  |  |  |  |

**Final remarks and thank you.**

Thank you very much for your participation. We will treat your personal data strictly confidential. If you have any questions or comments, please contact:

Sharmin Akter, ETH Zurich

Agricultural Economics and Policy Group

sakter@ethz.ch­­­­­­­­­­­­­­­­­­­­­­­­­­­­­­­­­­­­­­­­­­­­­­­­­­­­­­­­­­­­­­­­­­­­­­­­­­­­­­­­­­­­­­­

31. You said yes to receiving the results of the please let us know your email address so we can send you these results

32**.** Do you have any comments or feedback in general?

S4: Fact-sheet template used in the focus-group discussion in 2023 by each National Crop Clusters

| X [Title of the IPM tool] |
| --- |
| Write a short description of the IPM tool. How is it used? Why is it recommended? |
| X |
| When to apply/use the tool in the cropping cycle? |
| …............................................................................................................................................................................................. |
| Which biotic stress is the tool meant for: Diseases/Pests/Weeds/Other (plant growth regulation, …)? (Select one/more) |
| X |
| Could you specify the pest/disease/weeds? (Select one/more) |
| …............................................................................................................................................................................................. |
| To which of the three pillars of IPM does the tool belong? (Select one/more) |
| Prevention / Monitoring / Intervention |
| Which IPM principles are covered by the tool? (Select one/more of the 8 principles, listed in Table 1) |
| X |
| For intervention measures: what type of intervention? (Highlight the correct type) |
| - Chemical control (+ active substance + concentration/ha + frequency of application):   …...................................................................................................................................................   - Agronomic practices - Mechanical/Physical control - Biological control (beneficials, biopesticides) |
| Can this measure be used in organic cultivation? |
| Yes / No |
| Efficacy of the IPM tool against the relevant pest group(s): (choose 1-5; 1: very low efficacy against the relevant pest group (0-5%); 5: very high efficacy against the relevant pest group (50 % or more)) |
| X |
| Cost of implementation in labour time: (choose 1-5; 1: very little (0 - 0. 5 hours/ha); 5: very much (10 hours or more/ha)) |
| X |
| Cost of implementation in investments: (choose 1-5; 1: very low price (1% or less of the crop value/ha); 5: very high price (50% or more of the crop value/ha) |
| X |
| Performance (normal yield) compared to pesticide use: (choose 1-5; 1: negative impact (-5% or more); 5: positive impact (+5% or more) |
| X |
| Performance (standard quality) compared to pesticide use: (choose 1-5; 1: negative impact (-5% or more); 5: positive impact (+5% or more) |
| X |
| Potential reduction of pesticide use: (choose 1-5; 1: very low potential of reduction (almost 0%); 5: very high potential of reduction (25% or more) |
| X |
| Current level of implementation: (choose 1-5; 1: very low level of implementation (used on up to 5% of the cropped area); 5: very high level of implementation (used on 50% or more of the cropped area) |
| X |
| Level of 'readiness for use’:   - This tool is currently being used in practice. - This tool will be used in 1 – 5 years. - This tool will be used in 5 – 10 years.   Extra notes: |
